# Assessment of cardiac amyloidosis with ^99m^Tc-pyrophosphate (PYP) quantitative SPECT

**DOI:** 10.1186/s40658-020-00342-7

**Published:** 2021-01-07

**Authors:** Chao Ren, Jingyun Ren, Zhuang Tian, Yanrong Du, Zhixin Hao, Zongyao Zhang, Wei Fang, Fang Li, Shuyang Zhang, Bailing Hsu, Li Huo

**Affiliations:** 1 Department of Nuclear Medicine, Peking Union Medical College Hospital, Chinese Academy of Medical Science & Peking Union Medical College, Beijing Key Laboratory of Molecular Targeted Diagnosis and Therapy in Nuclear Medicine, Shuaifuyuan, Dongcheng District, Beijing, 100730 People’s Republic of China; 2 Department of Cardiology, Peking Union Medical College Hospital, Shuaifuyuan, Dongcheng District, Beijing, People’s Republic of China; 3 Department of Nuclear Medicine, Fuwai Hospital, Chinese Academy of Medical Science & Peking Union Medical College, Beijing, People’s Republic of China; 4 Nuclear Science and Engineering Institute, University of Missouri-Columbia, E2433 Lafferre Hall, Columbia, MO 65211 USA

**Keywords:** ATTR cardiomyopathy, ^99m^Tc-PYP quantitative SPECT, Standardized uptake value, Diagnostic feasibility, The operator reproducibility

## Abstract

**Background:**

^99m^Tc-PYP scintigraphy provides differential diagnosis of ATTR cardiomyopathy (ATTR-CM) from light chain cardiac amyloidosis and other myocardial disorders without biopsy. This study was aimed to assess the diagnostic feasibility and the operator reproducibility of ^99m^Tc-PYP quantitative SPECT.

**Method:**

Thirty-seven consecutive patients who underwent a ^99m^Tc-PYP thorax planar scan followed by SPECT and CT scans to diagnose suspected ATTR-CM were enrolled. For the quantitative SPECT, phantom studies were initially performed to determine the image conversion factor (ICF) and partial volume correction (PVC) factor to recover ^99m^Tc-PYP activity concentration in the myocardium for calculating the standardized uptake value (SUV) (unit: g/ml). SUV_max_ was compared among groups of ATTR-CM, AL cardiac amyloidosis, and other pathogens (others) and among categories of Perugini visual scores (grades 0–3). The intra- and inter-operator reproducibility of quantitative SPECT was verified, and the corresponded repeatability coefficient (RPC) was calculated.

**Results:**

The ICF was 79,327 Bq/ml to convert count rate in pixel to ^99m^Tc activity concentration. PVC factor as a function of the measured activity concentration ratio in the myocardium and blood-pool was [*y* = 1.424 × (1 − exp(− 0.759 × *x*)) + 0.104]. SUV_max_ of ATTR-CM (7.50 ± 2.68) was significantly higher than those of AL (1.96 ± 0.35) and others (2.00 ± 0.74) (all *p* < 0.05). SUV_max_ of grade 3 (8.95 ± 1.89) and grade 2 (4.71 ± 0.23) were also significantly higher than those of grade 1 (1.92 ± 0.31) and grade 0 (1.59 ± 0.39) (all *p* < 0.05). Correlation coefficient (*R*^2^) of SUV_max_ reached 0.966 to 0.978 with only small systematic difference (intra = − 0.14; inter = − 0.23) between two repeated measurements. Intra- and inter-operator RPCs were 0.688 and 0.877.

**Conclusions:**

^99m^Tc-PYP quantitative SPECT integrated with adjustable PVC factors is feasible to quantitatively and objectively assess the burden of cardiac amyloidosis for diagnosis of ATTR-CM.

## Background

Cardiac amyloidosis is related to the pathogen that the primary interstitial protein deposition occurs in the extracellular space of the myocardium, leading to impairment of myocardial wall contractility, systolic/diastolic dysfunction, arrhythmia, and eventually heart failure to cause high morbidity and mortality [1]. Main types of cardiac amyloidosis include monoclonal immunoglobulin light chain (AL) and transthyretin amyloidosis cardiomyopathy (ATTR-CM), of which ATTR-CM can be subtyped by pathogenic mutations in the transthyretin gene (ATTRm) or by the accumulation of amyloid fibrils composed of wild-type transthyretin protein (ATTRwt) [2–4]. In all different types of cardiac amyloidosis, the incidence rate varies between 5 and 13 per million per year [5–10]. Differential diagnosis of cardiac amyloidosis is often challenging. The most reliable approach to diagnose AL cardiac amyloidosis depends on blood and urine tests [11]. The traditional standard for diagnosis of cardiac ATTR amyloidosis relies on echocardiography (ECG) or cardiac magnetic resonance (CMR) along with that the deposit of cardiac amyloidosis should also be proved in an endomyocardial biopsy coupled with immunohistochemistry or mass spectroscopy [12, 13]. Nuclear medicine imaging can help to differentiate ATTR-CM from AL cardiac amyloidosis and other myocardial disorders without the need of biopsy. Positron emission tomography (PET) with β-amyloid-specific imaging tracers such as ^18^F-florbetapir, ^18^F-flutemetamol, and ^11^C-PIB enables the quantitative scheme to evaluate cardiac amyloidosis [14–16]. However, this quantitative imaging tool is not yet ready for routine clinical utilization. In recent years, systematic evaluation of the scintigraphy with ^99m^Tc-labeled phosphate tracers (e.g., technetium-99m 3, 3-diphospho-1, 2-propanodicarboxylic acid (^99m^Tc-DPD), technetium-99m pyrophosphate (^99m^Tc-PYP), or 99mTc-hydroxymethylene diphosphonate (^99m^Tc-HMDP)) has been reported as an outstanding non-invasive imaging tool to distinguish ATTR-CM from AL cardiac amyloidosis with excellent performance in differential diagnosis (sensitivity 84–97%, specificity 94–100%) [17–19]. Although the scintigraphy with ^99m^Tc-labeled phosphate tracers has demonstrated its effectiveness for diagnosis of ATTR-CM, there still exist relevant limitations in further identifying subgroups who may present different prognosis [20, 21]. The method of quantitative single-photon emission computed tomography (SPECT) has recently been developed to provide the quantitative assessment of amyloid burden adjunct to the visual interpretation of planar images. Several studies have further confirmed that quantitative SPECT possesses a potential in diagnosis of ATTR-CM independently [22–24]. The aim of our study is set to report the feasibility and the reproducibility of ^99m^Tc-PYP quantitative SPECT in differential diagnosis of cardiac amyloidosis when SPECT images were reconstructed with full physical corrections, and the correction for partial volume effect to recover true activity concentration in the myocardium was integrated into the quantitation process.

## Methods

### Study cohorts

Between December 2018 and December 2019, thirty-seven consecutive patients underwent a ^99m^Tc-PYP thorax planar scan followed by SPECT and CT scans to diagnose suspected ATTR-CM. For each study subject, routine examinations were carried out to record comprehensive clinical data accordingly. This study was complied with the amended Declaration of Helsinki approved by the Institutional Review Board of Peking Union Medical College Hospital. All participants provided the informed written consent. According to the previous research study [18], patients were divided into three groups primarily based on clinical features, immunohistochemical or proteomics typing of amyloid, ECG, Perugini visual scores, genetic analyses, and biopsy as the clinical routine for assessment of cardiac amyloidosis. Diagnosis of ATTR-CM included abnormal ECG finding and suggestive amyloidosis by visual grading of ^99m^Tc-PYP planar images equal to 2 or 3 with absence of a detectable monoclonal protein despite serum/urine immunofixation electrophoresis (IFE) and serum free light chain (sFLC) assay. Group A, ATTR-CM (*n* = 6), was based on clinical examination, ECG finding, positive ^99m^Tc-PYP finding in planar images with Perugini visual scores ≥ 2, and absence of abnormal serum/urine (IFE and sFLC). This diagnostic criterion identified one patient with ATTRwt and five patients with ATTRm. Heterogenous types of TTR mutations included Val50Gly (*n* = 1), Val50Met (*n* = 1), Gly73Glu (*n* = 1), Asp38Asn (*n* = 1), and Ala117Ser (*n* = 1). Group B, AL-CM (*n* = 10), was solely determined according to the presence of abnormal serum/urine (IFE or sFLC) as in lambda (λ)-light chain type (*n* = 7) and kappa (κ)-light chain type (*n* = 3). Group C, others (*n* = 21), is disqualified to fit into the diagnostic criteria of group A and group B. Several of them were ATTR mutation carriers from family history (*n* = 13) such as Ala117Ser (*n* = 7), Val50Met (*n* = 3), Ser97Tyr (*n* = 2), and Asp38Asn (*n* = 1) by genetic analyses but without evidence of showing the burden of cardiac amyloidosis. The remaining patients included hypertrophic cardiomyopathy (*n* = 2) and idiopathic cardiomyopathy (*n* = 5). Patient characteristics of these three groups are listed in Table 1.

**Table 1:** Patient characteristics

Groups	Total (*n* = 37)	Group A: ATTR cardiac amyloidosis (*n* = 6)	Group B: AL cardiac amyloidosis (*n* = 10)	Group C: others (*n* = 21)	*p* value
Demographics					
Age (years)	57.7 ± 13.2	58 ± 6	61 ± 3	56 ± 2	0.579
Male sex (%)	70.3	66.7	90.0	61.9	0.327
BMI (kg/m^2^)	23.7 ± 3.6	22.4 ± 1.7	22.7 ± 0.9	24.5 ± 0.8	0.256
Biopsy					
EMB (%)	16.2	16.7	40.0	4.8	0.028
Other tissue (%)	27.0	33.3	50.0	14.3	0.049
Laboratory					
Abnormal serum/urine IFE (%)	10.8	0.0	40.0	0.0	0.006
Abnormal sFLC (%)	24.3	0.0	90.0	0.0	< 0.0001
Echocardiography					
Abnormal (%)	10.8	83.3	80.0	19.0	< 0.0001
LVEF < 50% (%)	5.4	0.0	20	0.0	0.090
IVS or LVPW > 12 mm (%)	54.1	83.3	100.0	23.8	< 0.0001
Others					
Heart rate (beats per min)	82.2 ± 13.6	81.8 ± 3.0	83.9 ± 4.0	81.57 ± 3.4	0.908
Hypertension (%)	16.2	0.0	30.0	14.4	0.402

Continuous data are expressed as mean ± SD, and categorical data are expressed as percentages. *BMI* body mass index, *EMB* endomyocardial biopsy, *IFE* immunofixation electrophoresis, *sFLC* serum free light chain, *LVEF* left ventricle ejection fraction, *IVS* interventricular septal thickness, *LVPW* left ventricular posterior wall thickness

### Phantom experiment

For image quantitation, the experiment to derive the image conversion factor (ICF) to convert pixel value in quantitatively reconstructed SPECT images to ^99m^Tc activity concentration was initially conducted by filling ~ 740 MBq of ^99m^Tc water solution into a cylindrical phantom (radius 16 cm, height 20 cm). ICF was then calculated by the ^99m^Tc true activity concentration divided by pixel value. To note, ICF maintains a constant value when physical interference from attenuation, scatter, and statistical noise can be fully addressed in reconstructed images. To access partial volume effect (PVE) in the myocardium (Myo), a standard cardiac insert phantom (Data Spectrum Corporation, Hillsborough, NC, USA) representing a three-dimensional model of the left ventricle containing regions of the myocardial wall (~ 110 ml) and ventricle (~ 60 ml) was then utilized to measure PVE and to derive partial volume correction (PVC) factor under various activity concentration ratios (ACRs) of Myo and blood-pool (BP) in the ventricle cavity (0.15 to 10.0). PVE specified by the level of erroneous activity concentration was defined by the measured activity concentration divided by the true activity concentration in Myo. As reported, the degree of PVE considered as a function of wall thickness and Myo/BP ACR remains approximately a constant level in the circumstance that Myo/BP ACR is over a certain threshold, and inversely, it rises rapidly with the decreased Myo/BP ACR [25]. The unique property of PVE provides an opportunity to derive and fit the PVC factor (1.0/PVE) as an analytic function of Myo/BP ACR to recover the true activity concentration for the relatively unchanged wall thickness. In this study, acquisition parameters of SPECT scans for the phantom experiment were identical to those used in the patient scanning protocol as indicated in the next section.

### Image data acquisition

Each study subject was intravenously injected with a ~ 740-MBq ^99m^Tc-PYP dose prepared by Beijing SHIHONG Pharmaceutical Center and calibrated by a radioactivity meter (CRC-25R, CAPINTEC, USA). Relevant parameters including injection dose, time, and site were properly recorded. Post the ^99m^Tc-PYP injection for 1 h, a planar scan was performed in anterior and left lateral views for 10 min and then followed by a SPECT scan in the thorax position on a dual-head SPECT camera (Discovery 630, GE Healthcare, Haifa, Israel). The SPECT camera consists of low-energy high-resolution collimator with 9.53 mm thickness of NaI(Tl) scintillation crystal. With patient’s heart positioned in the center field of view, planar images were acquired for a total of 750,000 counts with 256 × 256 matrix and 1.46 zoom factor. Imaging parameters for SPECT acquisition utilized 128 × 128 matrix, circular orbit (radius 30 cm), 180° arc, step-and-shoot, 30 steps at 40 s/step, zoom = 1.0, and multiple energy windows (126–154 keV and 109–125 keV). After the completion of SPECT acquisition, a low-dose free-breathing CT scan (120 keV, 35 mA, 12 s) was separately acquired on a dedicated PET/CT scanner (Sinounion Polar Star m660, Beijing, China) for attenuation correction of SPECT images and image fusion. The patient positioning between two scans was optimally consistent to avoid non-translational misregistration.

### Image processing of quantitative SPECT

In this study, image reconstruction and data analysis of quantitative SPECT were performed using a cardiac software package (MyoFlowQ, Taipei, Taiwan). This software incorporates image reconstruction and subsequent image analysis on a single platform to measure ^99m^Tc or ^99m^Tc-PYP activity concentration in regions of myocardial wall and ventricle cavity. For the quantitative image reconstruction of SPECT, projection data were pre-corrected for ^99m^Tc isotope decay according to time points of rotation angles, and reconstructed by ordered subsets expectation maximization (OSEM) (4 iterations, 12 subsets) with full physical corrections for photon attenuation, scatter, collimator resolution, and Poisson count statistics as described previously [26–28]. Prior to the quantitative image reconstruction, a rapid image reconstruction with filtered back-projection (FBP) was preliminarily executed to provide quick SPECT images for the assessment of registration with CT images. SPECT-CT misregistration was verified visually and manually corrected by applying 3D translation to SPECT images. In the phantom experiment, a consistent region of interest (ROI) was drawn on SPECT images of the cylindrical phantom to count rate in pixel (unit: counts/second/pixel) to ^99m^Tc activity concentration (Bq/ml). To measure myocardial activity of the cardiac insert phantom, SPECT images were manually reoriented into the short-axis view. A threshold of 25% of peak activity was chosen to effectively differentiate between myocardial and ventricle regions. The myocardial centerline contour was automatically detected and refined by using an ellipsoid-approximated geometry with manually determined mitral valve plane to create the polar map. A consistent sampled region (1.0 × 1.0 × 2.0 cm^3^) was automatically placed in ventricle to measure the activity concentration of BP. PVC factor defined as the true ^99m^Tc activity concentration divided by the measured ^99m^Tc activity concentration from quantitative SPECT was presented in the scatter plot (*y*-axis = PVC factor, *x*-axis = measured Myo/BP ACR) and regressed with an exponential recovery model to derive analytic PVC factor as a function of measured Myo/BP ACR [29]. For the analysis of patients’ ^99m^Tc-PYP SPECT images, the same processing steps, including image reorientation, myocardial centerline contour, creation of polar map, and the placement of sampled region in ventricle cavity, were performed identically to those processing steps of cardiac insert phantom. Under the situation when the determination of myocardial centerline contour failed due to ultralow or no uptake of ^99m^Tc-PYP in the myocardium, a ROI (1.0 × 1.0 × 1.0 cm^3^) was manually placed in the insertion point between left and right ventricles on a transaxial plane of reconstructed images as the located joint of apex in the right ventricle with the apical septal of left ventricle. Post the recovery to absolute ^99m^Tc-PYP uptake using PVC factor derived from the phantom experiment, standardized uptake value (SUV) was calculated with factors of injected ^99m^Tc-PYP dose and patient’s body weight.

### Interpretation of planar images and semi-quantitative measurement

Both anterior and lateral views of ^99m^Tc-PYP planar images were evaluated by two consensus nuclear readers in nuclear cardiology to grade using the visual grading rule reported by Perugini et al. as follows: grade 0 = cardiac uptake not visible, grade 1 = mild cardiac uptake visible but inferior to skeletal uptake, grade 2 = moderate cardiac uptake visible equal to or greater than skeletal uptake, and grade 3 = strong cardiac uptake with little or no skeletal uptake. The semi-quantitative analysis of planar images was performed by drawing a patient-specific circular ROI on the heart and mirroring it to the contralateral chest in order to calculate the heart-to-contralateral (H/CL) ratio from the quotient of the mean counts [17].

### Measurement of intra- and inter-operator reproducibility

Correlations of image processing for semi-quantitative and quantitative parameters by the 1st operator (OP1) and the 2nd operator (OP2) were verified by linear regression. OP1 had 20 years of experience in image processing of nuclear cardiology, and OP2 encompassed 3 years of experience. To test the intra-operator reproducibility, OP1 processed all image data twice in 4 weeks apart. To test the inter-operator reproducibility, OP2 processed the same image datasets independently.

### Statistics analysis

All datasets were analyzed with a statistical software package (IBM SPSS Statistics version 25). Continuous variables were presented as mean ± SD, whereas categorical variables were expressed as actual numbers and percentage. For the comparison between study subgroups, differences in continuous variables were analyzed using the one-way ANOVA with post hoc Bonferroni correction when Levene’s pre-test for homogeneity of variances meets the requirement; otherwise, the one-way Welch ANOVA with post hoc Games-Howell correction was applied. Differences in categorical variables were analyzed using the *χ*^2^ test or Fisher exact test. The correlation of H/CL ratio and quantitative parameter (SUV_max_) from OP1 and OP2 was obtained by the linear regression. Difference of correlation coefficient between two measurements was tested by the *Z* test. Bland-Altman statistics were utilized to verify the systematic difference with a 95 % confidence interval (CI) for semi-quantitative and quantitative parameters. The repeatability coefficient (RPC) representing intra- and inter-operator reproducibility was calculated as RPC = 1.96 × SD of difference between the two measurements [30]. All *p* values used were two sided with *p* < 0.05 considered statistically significant.

## Results

### Phantom experiment

**Fig. 1:**
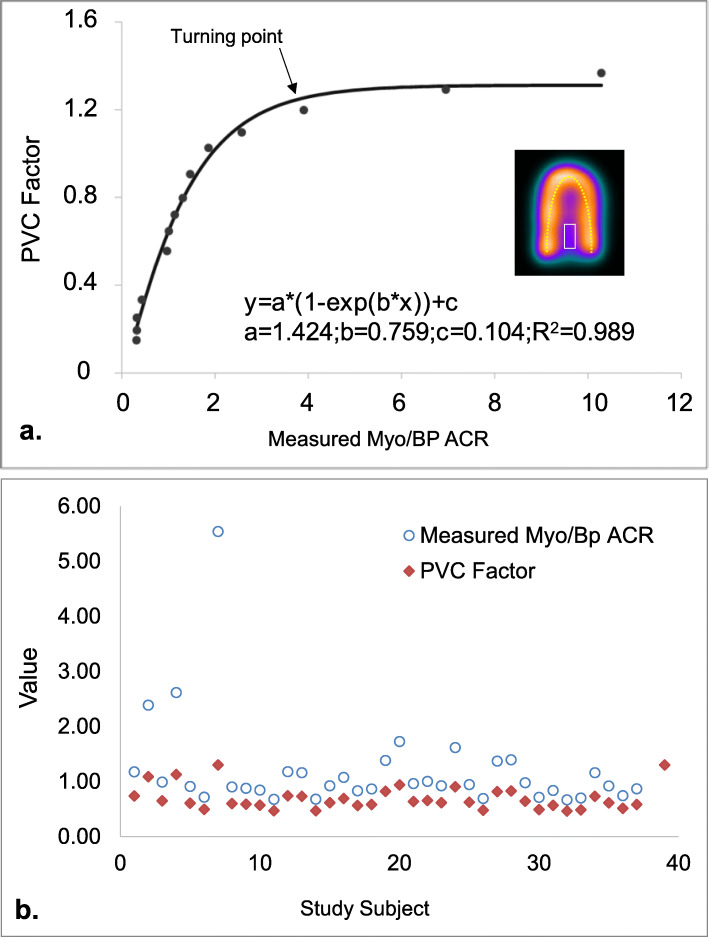
**a** PVC factor to recover as a function of measured activity concentration ratio (ACR) in Myo and BP regions. **b** Measured Myo/BP ACR and corresponded PCV factor for individual study subject

Through the cylindrical phantom experiment, the ICF to convert the pixel value to the corresponded activity concentration in quantitative SPECT images was 79,327 Bq/ml per cps/pixel. Activity concentrations in Myo and BP regions were measured in the unit of becquerels per milliliter and then applied to derive PVC factor. Experimental data points of Myo/BP ACR and corresponded PVC factors were (0.315, 0.149), (0.324, 0.194), (0.428, 0.334), (0.967, 0.334), (0.967, 0.646), (1.136, 0.720), (1.307, 0.796) (1.464, 0.906), (1.858, 1.026), (2.571, 1.097), (3.903, 1.198), (6.952, 1.291), and (10.28, 1.367). Figure 1a shows the scatter plot of PVC factor vs the measured Myo/BP ACR. While the data of scatter plot were further fitted with an exponential recovery model, a strong correlation coefficient (*R*^2^) as 0.998 was observed to generate an analytic curve as: *y* = *a* × (1 − exp(− *b* × *x*)) + *c*, where parameters *a*, *b*, and *c* were 1.424, 0.759, and 0.104, respectively. In the curve, the PVC factor stayed as a constant (1.31) when the measured Myo/BP ACR was ≥ 4.0 (PVC = 1.260 as 95.4% of 1.31), and it declined dramatically below the turning point.

### ^99m^Tc-PYP image findings

**Fig. 2:**
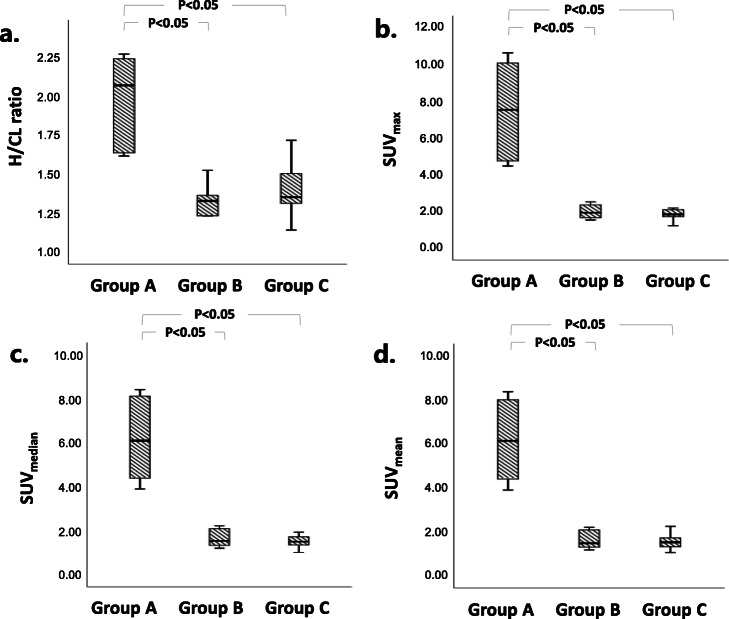
The box plots of H/CL ratio, SUV_max_, SUV_median_, and SUV_mean_ among ATTR-CM (group A), AL cardiac amyloidosis (group B), and others (group C)

**Fig. 3:**
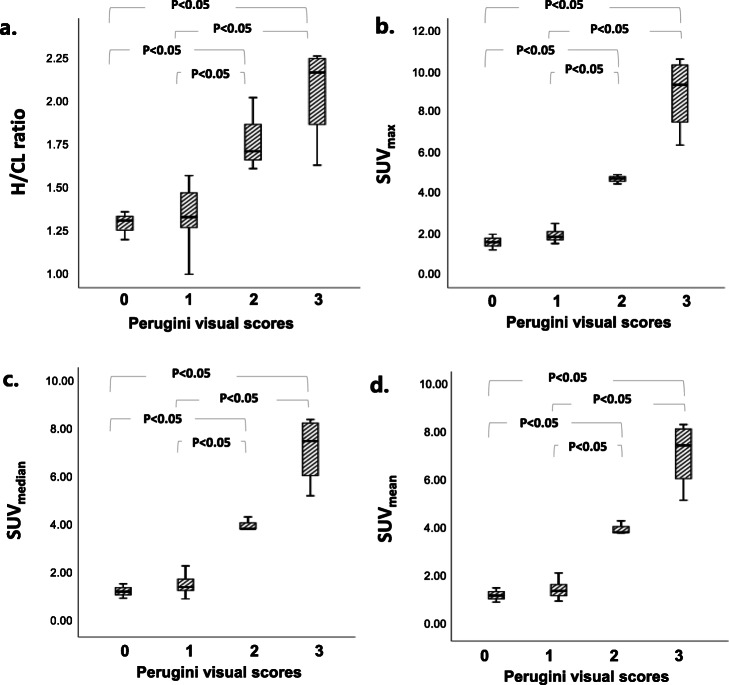
The box plots of H/CL ratio, SUV_max_, SUV_median_, and SUV_mean_ among groups of Perugini visual scores (grades 0–3)

**Fig. 4:**
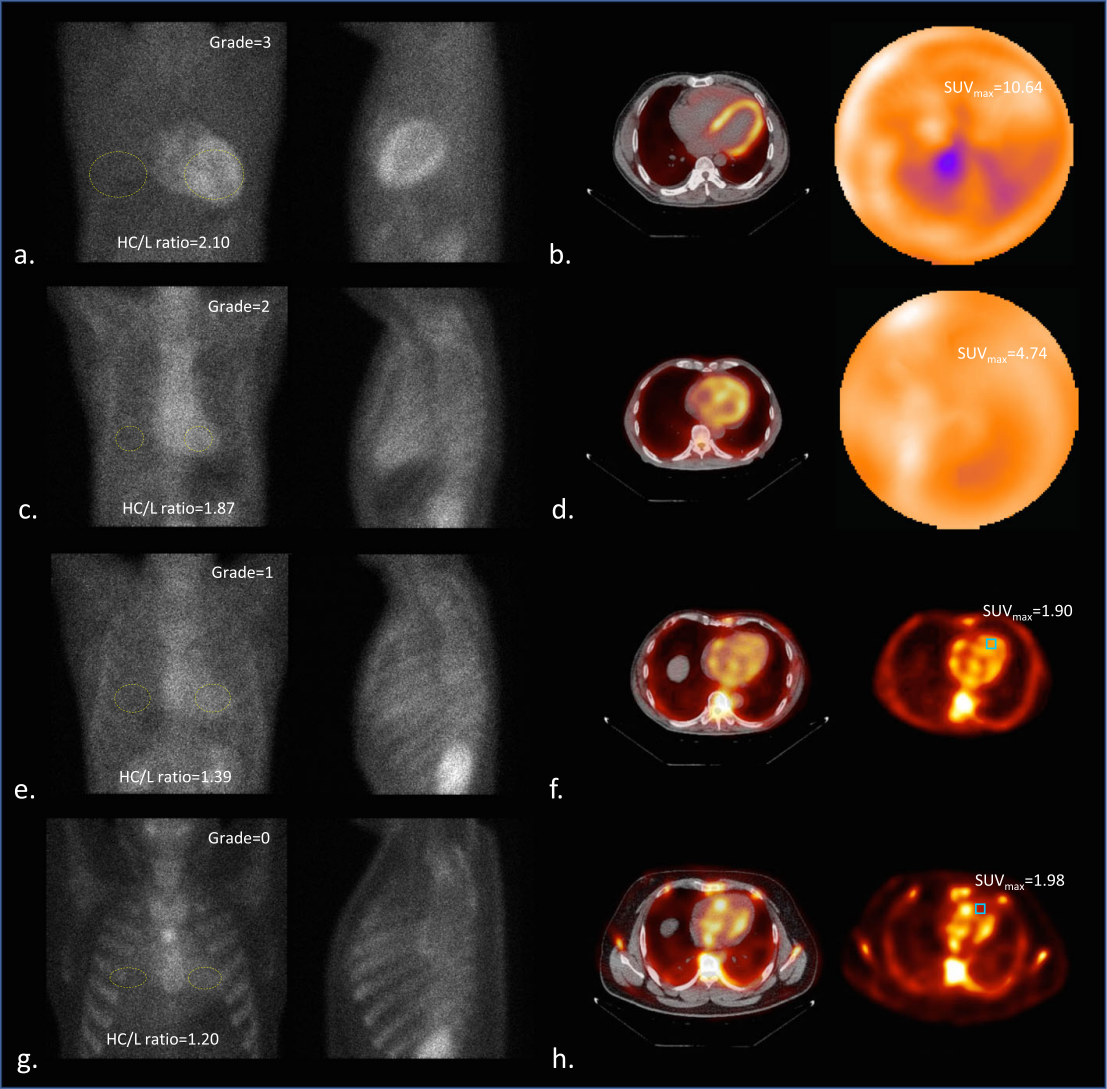
Representative images of ^99m^Tc-PYP planar and quantitative SPECT. **a** Grade = 3 in Perugini visual scores with H/CL ratio = 2.10 and SUV_max_ = 10.64 g/ml in **b**. **c** Grade = 2 with H/CL ratio = 1.87 and SUV_max_ = 4.74 g/ml in **d**. **e** Grade = 1 with H/CL ratio = 1.39 and SUV_max_ = 1.90 g/ml in **f**. **e** Grade = 0 with H/CL ratio = 1.20 and SUV_max_ = 1.98 g/ml in **h**

Image findings of planar and quantitative SPECT for diagnosed ATTR-CM, AL cardiac amyloidosis, and others are summarized in Table 2. In group A diagnosed as ATTR-CM, 66.7% had Perugini scores = 3 and 33% for Perugini visual scores = 2 while 100% of group B diagnosed as AL cardiac amyloidosis had Perugini visual scores = 1. In group C diagnosed by other pathogens, Perugini visual scores were dispersed from 0 to 2. From ^99m^Tc-PYP planar images, H/CL ratio of group A (1.98 ± 0.29) was significantly higher than those of group B (1.28 ± 0.16) and group C (1.38 ± 0.19) (*p* < 0.0001). From ^99m^Tc-PYP quantitative SPECT, the measured Myo/BP ACR ranges from 0.665 to 5.542 to give PVC factor from 0.46 to 1.30 (Fig. 1b). With the recovery of activity concentration in the myocardium for all study subjects using PVC factor derived from the cardiac phantom experiment, SUV_max_ of group A (7.50 ± 2.68 g/ml) was also significantly higher than those of group B (1.96 ± 0.35) and group C (2.00 ± 0.74) (all *p* < 0.05). Similar findings were observed for SUV_median_ and SUV_mean_ as listed in Table 2. Figure 2 shows the box plots of H/CL ratio, SUV_max_, SUV_median_, and SUV_mean_ for the pathological groups. From the semi-quantitative analysis of ^99m^Tc-PYP planar images, grades 0 and 1 had significantly lower H/CL ratio (1.29 ± 0.81 and 1.34 ± 0.18) than grades 2 and 3 (1.78 ± 0.21 and 2.06 ± 0.29). Furthermore, there was no significant difference between either grades 0 and 1 or grades 2 and 3 (all *p* < 0.05). From ^99m^Tc-PYP quantitative SPECT, SUV_max_ of grade 3 (8.95 ± 1.89 g/ml) and grade 2 (4.71 ± 0.23) were significantly higher than those of grade 1 (1.92 ± 0.31) and grade 0 (1.59 ± 0.39) (all *p* < 0.05). Additionally, neither difference between grades 3 and 2, nor difference between grades 1 and 0 was significant. Findings of planar and quantitative SPECT categorized by Perugini visual scores (grades 0 to 3) are summarized in Table 3 and plotted in box plots in Fig. 3. Figure 4 shows representative patients with planar images acquired in anterior and lateral views to measure H/CL ratio and corresponded quantitative SPECT images to derive SUV_max_ in the myocardium.

**Table 2:** ^99m^Tc-PYP findings from planar and quantitative SPECT

Groups	Total (*n* = 37)	Group A: ATTR cardiac amyloidosis (*n* = 6)	Group B: AL cardiac amyloidosis (*n* = 10)	Group C: others (*n* = 21)	*p* value
Planar					
Visual scores (%)					
0	8.1	0	0	14.3	< 0.0001
1	73	0	100	81	
2	8.1	33.3	0	4.8	
3	10.8	66.7	0	0	
H/CL ratio	1.45 ± 0.31	1.98 ± 0.29	1.28 ± 0.16^*^	1.38 ± 0.19^*^	< 0.0001
Quantitative SPECT					
SUV_max_	2.88 ± 2.36	7.50 ± 2.68^^,^^^	1.96 ± 0.35	2.00 ± 0.74	0.002
SUV_median_	2.38 ± 1.90	6.17 ± 1.92^^,^^^	1.65 ± 0.39	1.65 ± 0.60	0.001
SUV_mean_	2.36 ± 1.90	6.16 ± 1.90^^,^^^	1.61 ± 0.40	1.63 ± 0.60	0.001
SUV_bone_	1.09 ± 0.29	1.17 ± 0.34	1.28 ± 0.22^**^	0.97 ± 0.25	0.01

Continuous data are expressed as mean ± SD, and categorical data are expressed as percentages

^*^p < 0.05 by ANOVA with Bonferroni correction in comparison to Group A

^**^p < 0.05 by ANOVA with Bonferroni correction in comparison to Group C

^^^*p* < 0.05 by Welch ANOVA with Games-Howell correction in comparison to Group B

^^^^*p* < 0.05 by Welch ANOVA with Games-Howell correction in comparison to Group C

**Table 3:** ^99m^Tc-PYP findings from planar and quantitative SPECT/CT images for groups divided by Perugini visual scores

Groups	Total (*n* = 37)	Grade 0 (*n* = 3)	Grade 1 (*n* = 27)	Grade 2 (*n* = 3)	Grade 3 (*n* = 4)	*p* value
Planar						
H/CL ratio	1.45 ± 0.31	1.29 ± 0.81^*,**^	1.34 ± 0.18^*,**^	1.78 ± 0.21	2.06 ± 0.29	< 0.0001
Quantitative SPECT/CT						
SUV_max_	2.90 ± 2.35	1.59 ± 0.39^^,^^^	1.92 ± 0.31^^,^^^	4.71 ± 0.23	8.95 ± 1.89	< 0.0001
SUV_median_	2.41 ± 1.90	1.33 ± 0.30^^,^^^	1.60 ± 0.34^^,^^^	4.06 ± 0.28	7.18 ± 1.43	< 0.0001
SUV_mean_	2.39 ± 1.90	1.34 ± 0.30^^,^^^	1.57 ± 0.33^^,^^^	4.08 ± 0.28	7.16 ± 1.39	< 0.0001

Continuous data are expressed as mean ± SD, and categorical data are expressed as percentages

^*^p < 0.05 by ANOVA with Bonferroni correction in comparison to grade 2

^**^p < 0.05 by ANOVA with Bonferroni correction in comparison to grade 3

^^^*p* < 0.05 by Welch ANOVA with Games-Howell correction in comparison to grade 2

^^^^*p* < 0.05 by Welch ANOVA with Games-Howell correction in comparison to grade 3

### Intra- and inter-operator reproducibility

**Fig. 5:**
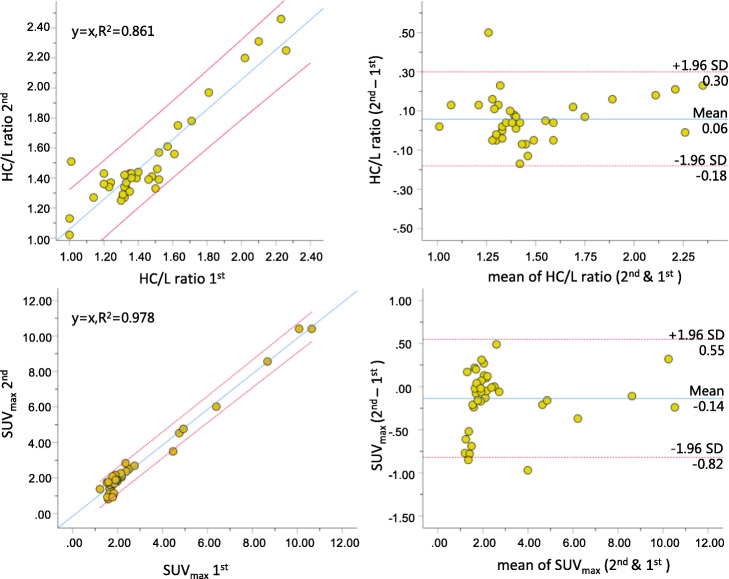
Linear regression and Bland-Altman plots of H/CL ratio and SUV_max_ measured by OP1 who processed the same image sets in 4 weeks apart

**Fig. 6:**
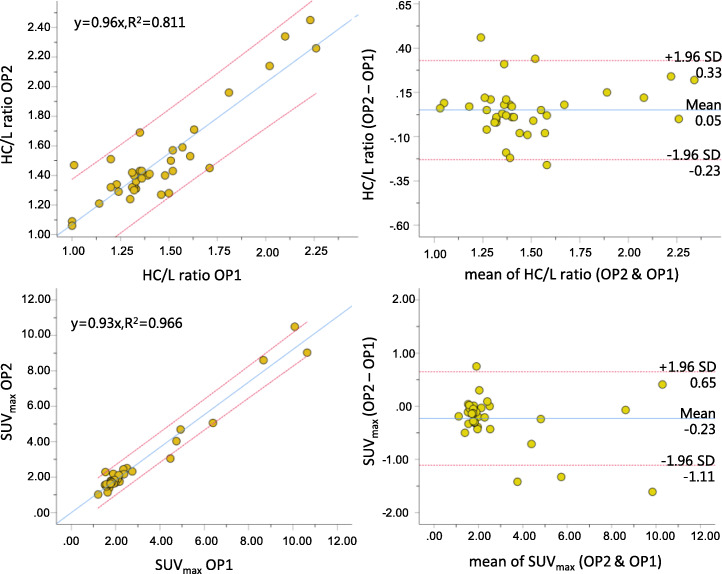
Linear regression and Bland-Altman plots of H/CL ratio and SUV_max_ measured by OP1 and OP2 who processed the same image sets independently

Figure 5 shows the linear regression and Bland-Altman plots of H/CL ratio and SUV_max_ from the OP1 who processed ^99m^Tc-PYP planar and quantitative SPECT images twice in 4 weeks apart. The linear regression demonstrated that excellent corrections existed for OP1 to process H/CL ratio (*R*^2^ = 0.861) and SUV_max_ (*R*^2^ = 0.978) repeatedly. Differences in correlation coefficients for either H/CL ratio or SUV_max_ (*Z* scores = − 3.921, *p* < 0.0001) were significant. From the Bland-Altman plots, mean difference of H/CL was 0.06 (95% CI = − 0.18–0.30) and determined as − 0.14 (95% CI = − 0.82–0.55) for SUV_max_. Values of the intra-operator RPC for H/CL ratio and SUV_max_ were 0.241 and 0.688 g/ml, respectively. Figure 6 shows the linear regression and Bland-Altman plots of H/CL ratio and SUV_max_ from OP1 and OP2 who processed ^99m^Tc-PYP planar and quantitative SPECT images independently. The linear regression demonstrated that excellent correlation also existed for OP1 and OP2 to process H/CL ratio (*R*^2^ = 0.811) and SUV_max_ (*R*^2^ = 0.966). Differences in correlation coefficients for H/CL ratio and SUV_max_ were significant (*Z* scores = − 0.3716, *p* = 0.0002). From the Bland-Altman plots, mean difference of H/CL was − 0.05 (95% CI = − 0.23–0.33) and determined as − 0.23 (95% CI = − 1.11–0.65) for SUV_max_. Values of the inter-operator RPC for H/CL ratio and SUV_max_ were 0.280 and 0.877 g/ml.

## Discussion

We initially conducted the phantom study to obtain the ICF for quantitative SPECT and to derive PVC factor for recovering true activity concentration in the myocardium. To note, the PVC factor described by fitting parameters (*a*, *b*, *c*) in the exponential recovery function can only be transferable to the same camera system with the matched set of imaging parameters (e.g., matrix and pixel size). To obtain PVC factor for different camera models, dissimilar sets of imaging parameters, or both, the described phantom experiment and data fitting process should be reperformed to warrant an appropriate recovery function. Indeed, the unique characteristic of PVC factor curve as a function of the measured Myo/BP ACR elucidated that the impact to the myocardium coming from the activity in surrounded area is rather a constant, but varied depending on Myo/BP ACR. To resolve this issue is particularly important for ^99m^Tc-PYP quantitative SPECT as the diagnosis for ATTR-CM must rely on accurate activity measurement. Nonetheless, the solution is not yet applicable in current commercial software packages, therefore hampering ^99m^Tc-PYP quantitative SPECT as a clinical utility [23, 24]. In our study with a majority of patients (29/37) presenting low Myo/BP ACR, we found PVC factor varied in a large range from 0.46 to 1.30 (Fig. 1b). By using individual’s adjustable PVC factor based on the recovery curve, we found SUV_max_, SUV_median_, and SUV_mean_ were able to differentiate the ATTR-CM group from groups of AL cardiac amyloidosis and others. For the same cohorts further categorized by Perugini visual scores, SUV_max_, SUV_median_, and SUV_mean_ were able to distinguish groups of grades 2 and 3 from grades 0 and 1. ^99m^Tc-PYP quantitative SPECT integrated with adjustable PVC factors is therefore feasible to quantitatively assess the burden of cardiac amyloidosis for diagnosis of ATTR-CM. In our study, we further evaluated the reproducibility of ^99m^Tc-PYP quantitative SPECT. The intra- and inter-reproducibility of the quantitative method were excellent as *R*^2^ reached 0.902 to 0.978 with only small systematic difference (intra = − 0.14 to − 0.06; inter = − 0.23 to − 0.11) between two repeated measurements. Both intra- and inter-reproducibility outperformed that of the semi-quantitative method (*R*^2^ 0.811–0.861, all *p* < 0.0267). ^99m^Tc-PYP quantitative SPECT developed in this study can be a reproducible and reliable method to measure quantitative parameters (e.g., SUV). To our knowledge, this is the first study to provide the relevant information.

The most widely used gage for ^99m^Tc-PYP scintigraphy for differentiating ATTR-CM from AL cardiac amyloidosis is developed by Perugini via a visual comparison of the myocardium to ribs [20]. Although Perugini visual scoring method is simple and straightforward to implement clinically, the downside of this method is still marked as highly subjective among readers [31]. The semi-quantitative analysis with H/CL has been developed to improve the objectivity effectively. However, the method still suffered from low subjectivity by the manual drawing of ROI on myocardial and CL regions. Under the circumstance of intense extra-cardiac uptake or background, the diagnostic certainty of both methods can no longer be preserved [32]. As proposed, absolute quantitation of myocardial uptake using quantitative SPECT provides a solution to improve objectivity and reliability for ^99m^Tc-PYP scintigraphy [23]. In our study, we demonstrated that the quantitative SPECT integrated with adjustable PVC factors is useful as not only a savior to visual interpretation on planar images suffering from intense extra-cardiac uptake or myocardial uptake overlapped with blood-pool or bone, but also a quantitative and objective imaging method to measure the burden of amyloid deposit in the myocardium. Related patient examples are shown in Fig. 4.

Previous studies reported that images produced by ^99m^Tc-labeled phosphate tracers (e.g., ^99m^Tc-DPD, ^99m^Tc-PYP, ^99m^ Tc-HMDP) are not actually identical for the diagnosis of ATTR-CM [33]. The pattern of increased SUV_max_ in the group of grade = 3 vs the group of grade = 2 by Perugini visual scores was observable for ^99m^Tc-PYP, but not for ^99m^Tc-DPD or ^99m^ Tc-HMDP [24]. This exceptional difference may provoke an additional value in prognosis for high grade of ATTR-CM with ^99m^Tc-PYP quantitative SPECT. Recently, quantitative PET with bone scan agent, ^18^fluorine-labeled sodium fluoride (^18^F-NaF), was evaluated for the diagnosis of ATTR-CM, but not quite promising [34]. Quantitative PET with β-amyloid-specific imaging tracers such as ^18^F-florbetapir, ^18^F-flutemetamol, and ^11^C-PIB enabled the quantitative scheme to evaluate cardiac ATTR amyloidosis [14–16]. However, this PET quantitative imaging tool for diagnosis with a potential in prognosis of AL amyloidosis is not yet ready for routine clinical utilization [35]. As ^99m^Tc-labeled phosphate tracers are widely available, quantitative SPECT can be valuable for the diagnosis for ATTR-CM and potentially for the prognosis. Moreover, it can provide a quantitative tool to monitor the disease progression for individual with ATTR mutation carriers from family history who does not yet present clinically relevant symptoms. It also enables quantitative assessment of treatment response to proven therapy as well as helpful in conducting trials of new therapeutic agents.

### Study limitations

In this study, only a limited sample size for the ATTR-CM (*n* = 6) group was available. This limitation restricted to further statistically differentiate the group of Perugini visual scores = 3 from the group of Perugini visual scores = 2 by using the quantitative parameter, SUV_max_, although the pattern was observable as shown in our data. Future study should focus to resolve this limitation by increasing the sample size of the ATTR group. Another limitation is that no prognosis data were available. Whether SUV_max_ or the heterogeneity of SUV_max_ can provide better prognosis than Perugini visual scores or H/CL ratio cannot be answered by this study. Other related technical limitation may be addressed by SPECT and CT data acquired on separate scanners. When non-translational misregistration between SPECT and CT images (e.g., rotational misregistration) may occur, the current program cannot compensate to correct for the type of error. Nonetheless, in our study, no study subject actually showed non-translational misregistration when careful patient positioning between SPECT and CT scans was carried out. Other limitation may be addressed that this study only validated the inter- and intra-operator reproductivity for the quantitative SPECT with a single ^99m^Tc-PYP scan, and did not provide relevant data to verify the reproductivity among multiple ^99m^Tc-PYP scans on the same cohort.

## Conclusions

^99m^Tc-PYP quantitative SPECT integrated with adjustable PVC factors is feasible to quantitatively and objectively assess the burden of cardiac amyloidosis for diagnosis of ATTR-CM.

## Data Availability

The datasets used and/or analyzed during the current study were available from corresponding author on reasonable request.

## Abbreviations

ACR: Activity concentration ratio
AL: Light chain amyloidosis
ATTR: Amyloid transthyretin
ECG: Echocardiography
EMB: Endomyocardial biopsy
FBP: Filtered back-projection
H/CL: Heart-to-contralateral
HFpEF: Heart failure with ejection fraction
ICF: Image conversion factor
IFE: Immunofixation electrophoresis
IVS: Interventricular septal
LVEF: Left ventricle ejection fraction
LVPW: Left ventricular posterior wall
OSEM: Ordered subsets expectation maximization
PET: Positron emission tomography
PVC: Partial volume correction
PYP: Pyrophosphate
RPC: Repeatability coefficient
sFLC: Serum free light chain
SPECT: Single-photon emission computed tomography
SUV: Standardized uptake value
^99m^Tc: ^99m^Technetium
